# Phosphoinositide Lipids in Ocular Tissues

**DOI:** 10.3390/biology9060125

**Published:** 2020-06-12

**Authors:** Ammaji Rajala, Austin McCauley, Richard S. Brush, Khuong Nguyen, Raju V.S. Rajala

**Affiliations:** 1 Departments of Ophthalmology, University of Oklahoma Health Sciences Center, Oklahoma City, OK 73104, USA; ammaji-rajala@ouhsc.edu (A.R.); Austin-McCauley@ouhsc.edu (A.M.); Richard-brush@ouhsc.edu (R.S.B.); khuong-nguyen@ouhsc.edu (K.N.); 2 Dean McGee Eye Institute, University of Oklahoma Health Sciences Center, Oklahoma City, OK 73104, USA; 3 Departments of Cell Biology, University of Oklahoma Health Sciences Center, Oklahoma, OK 73104, USA; 4 Department of Physiology, University of Oklahoma Health Sciences Center, Oklahoma City, OK 73104, USA

**Keywords:** phosphoinositides, cellular signaling, retina, retinal pigment epithelium, cornea

## Abstract

Inositol phospholipids play an important role in cell physiology. The inositol head groups are reversibly phosphorylated to produce seven distinct phosphorylated inositides, commonly referred to as phosphoinositides (PIs). These seven PIs are dynamically interconverted from one PI to another by the action of PI kinases and PI phosphatases. The PI signals regulate a wide variety of cellular functions, including organelle distinction, vesicular transport, cytoskeletal organization, nuclear events, regulation of ion channels, cell signaling, and host–pathogen interactions. Most of the studies of PIs in ocular tissues are based on the PI enzymes and PI phosphatases. In this study, we examined the PI levels in the cornea, retinal pigment epithelium (RPE), and retina using PI-binding protein as probes. We have examined the lipids PI(3)P, PI(4)P, PI(3,4)P_2_, PI(4,5)P_2_, and PI(3,4,5)P_3_, and each is present in the cornea, RPE, and retina. Alterations in the levels of these PIs in mouse models of retinal disease and corneal infections have been reported, and the results of our study will help in the management of anomalous phosphoinositide metabolism in ocular tissues.

## 1. Introduction

**Figure 1:**
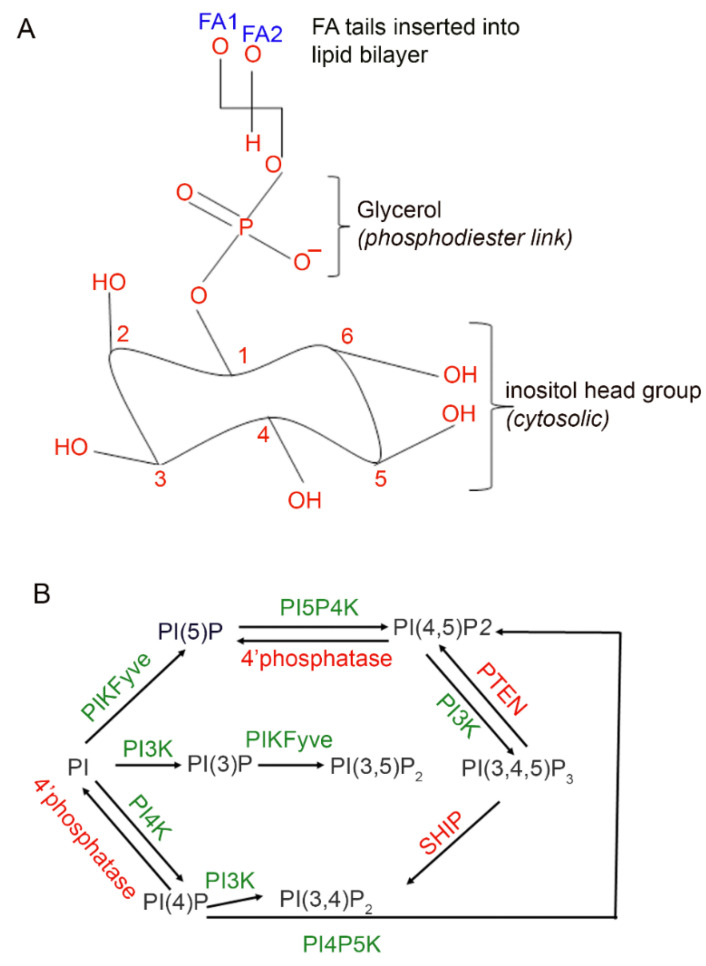
Seven distinct phosphoinositide molecules in the cell. The parent phosphoinositide (PI) contains a myo-inositol head group, and a glycerol backbone, and two fatty acids (FA1 and FA2) are attached to the C1 and C2 position of the glycerol (**A**). The free hydroxyl group, 3, 4, 5 on the inositol ring undergoes phosphorylation by PI kinases (green). Dephosphorylation by PI phosphatases (red) generates seven distinct phosphoinositide second messenger molecules (black) in the cell (**B**). PI, phosphatidylinositol; PI(3)P, phosphatidylinositol 3-phosphate; PI(4)P, phosphatidylinositol 4-phosphate; PI(5)P, phosphatidylinositol 5-phosphate; PI(3,4)P_2_, phosphatidylinositol 3,4-bisphosphate; PI(4,5)P_2_, phosphatidylinositol 4,5-bisphosphate; PI(3,5)P_2_, phosphatidylinositol 3,5-bisphosphate; PI(3,4,5)P_3_, phosphatidylinositol 3,4,5-trisphosphate; PI3K, phosphoinositide 3-kinase; PI4K, phosphoinositide 4-kinase; PI4P5K, phosphatidylinositol 4-phosphate 5-kinase; PI5P4K, phosphatidylinositol 5-phosphate 4-kinase; PTEN, phosphatase and tensin homolog; SHIP, Src homology 2 (SH2) domain-containing inositol polyphosphate 5-phosphatase; PIKfyve, phosphatidylinositol-3-phosphate 5-kinase type III or PIPKIII or FYVE phosphoinositide kinase.

Phosphoinositides are low-abundance lipids and constitute ~0.5% to 1% of the total phospholipid pool. The parent phosphatidylinositol (PI) molecule contains a myo-inositol head group with a glycerol backbone and two fatty acids attached to the C1 and C2 positions of the glycerol [1,2,3,4]. The free hydroxyl groups on the inositol ring undergo phosphorylation by PI kinases; the phosphorylation products are called phosphoinositides [2]. By the action of PI kinases and PI phosphatases, seven distinct phosphoinositide signaling molecules have been identified [1,2] (Figure 1). These seven distinct phosphoinositide signals regulate various cellular events, including vesicular trafficking, cytoskeletal organization, membrane fusion, phagocytosis, and cell survival [1,2,3].

The eye is the main organ for visual perception. The retina is neuronal tissue composed of several neurons and one epithelial cell type, the retinal pigment epithelium (RPE). The cornea is located in the front of the eye and is a clear surface of the eye. Light passes through the cornea and the image is processed in the retina, whereas the RPE provides nourishment to the retina. The presence of PI signaling pathways, mainly the PI kinases and PI phosphatases in the cornea [5], retina, and RPE [3,6], has been reported previously. In the retina, the PI signaling pathway is light-dependent, and most of the enzymes of the PI cycle are activated by light [6,7,8,9]. The PI levels in rod outer segments and the role of PI(3)P in rods, bipolar cells, and RPE have been reported [6,10,11,12,13]. Earlier studies showed that PI lipids play important roles in phototransduction [14,15], protein trafficking [16,17], and channel modulation [18,19]. Compared with other areas, few studies on PI signaling in the ocular tissues have been reported.

During viral infection, pathogens hijack the PI pathways and use them for entry and survival in the host cell [20,21,22]. Consistent with these studies, we previously reported that corneal cells infected with Adenovirus 19 utilize class I phosphoinositide 3-kinase/Akt activation for viral pathogenesis [23]. The Class I PI3-kinase (PI3K) signaling pathway is one of the most important PI-mediated signaling cascades activated during virus entry. Activation of PI3K and subsequent generation of PI(3,4,5)P_3_, which serves as a docking platform for proteins carrying lipid-binding domains, including Akt, the main effector of PI3K signaling [24].

While PI3K is involved in all forms of endocytosis, its best-characterized role is in macropinocytosis. PI3K serves to co-ordinate signaling and cytoskeletal modulation during the protrusion, extension, and closure phases of macropinosome formation [24]. Activation of PI3K upon virus binding has been observed for several viruses that use this entry mechanism, including influenza, Herpes simplex virus type 1 (HSV-1), HCV, Zaire Ebola Virus (ZEBOV), vaccinia virus (VACV) [24], and Zika virus [25]. The collective evidence indicates that activation of the PI3K signaling pathway is a common strategy utilized by multiple viruses to different ends [24].

The tubby domain of tubby and tubby-like proteins (TULP) proteins has a high affinity for binding to PI(4,5)P_2_ [26]. The tubby domain in tubby protein binds to both DNA and PI [26]. Mutations in the *TULP1* gene cause recessive retinitis pigmentosa [27,28]. The PIKfyve gene encodes an enzyme, PIKfyve, also known as phosphatidylinositol-3-phosphate 5-kinase type III or PIPKIII), which phosphorylates PI to PI(5)P as well as phosphorylates PI(3)P to PI(3,5)P_2_ [29]. Mutations in one of the alleles of PIKfyve are linked to Francois–Neetens corneal fleck dystrophy [30]. PIKfyve plays an important role in generating PI(5)P upon binding to PI-3-P through its FYVE domain [29]. This PI(5)P can be converted to PI(4,5)P_2_ through the action of the type II phosphatidylinositol 5-phosphate 4-kinase (PIP4K) enzyme, which was previously shown to express in rod photoreceptor cells [31]. Congenital cataracts are formed owing to mutations in the FYCO1 (FYVE and coiled-coli domain autophagy adapter 1) gene [32], a PI(3)P binding protein [33]. The 5’PI phosphatase INPP5E is coupled with proper ciliogenesis [34,35]. INPP5E dephosphorylates PI(3,4,5)P_3_ to PI(4,5)P_2_ [35]. This inositol phosphatase is important in ciliary development in zebrafish [36]. Mutations in INPP5E result in Joubert syndrome, a rare disorder characterized by deformation of the midbrain, retinitis pigmentosa, renal cysts, and polydactyly [37]. There was an excellent review published this year on the role of phosphoinositides in retinal function and disease [13]. In this study, we used PI-binding probes to examine the PI lipids in the cornea, RPE, and photoreceptor cells. This approach will help us to measure these lipids in disease states and modulate these lipids under disease conditions for therapeutic benefits.

## 2. Materials and Methods

### 2.1. Materials

The rhodopsin 1D4 antibody was a kind gift from Dr. James F. McGinnis (OUHSC). The amylose affinity resin was obtained from New England Bio labs (Ipswich, MA, USA). All other reagents were of analytical grade and purchased from Sigma (St. Louis, MO, USA). Glutathione agarose beads were obtained from ThermoFisher Scientific (Waltham, MA, USA). PIP strips, PI(3)P, PI(4)P, PI(5)P, PI(3,4)P_2_, PI(4,5)P_2_ and PI(3,4,5)P_3_ lipids, PI3-kinase alpha (PI3Kα) active enzyme, and PTEN enzyme were obtained from Echelon Biosciences (Salt Lake City, UT, USA).

### 2.2. Methods

#### 2.2.1. Isolation of Rod Outer Segments by Discontinuous Sucrose Density Gradient Centrifugation

Bovine eyes were obtained from a local abattoir and the retinas were dissected and placed on ice. Rod outer segments (ROS) were prepared using discontinuous sucrose density centrifugation. Retinas were placed in a glass/PTFE Potter–Elvehjem pestle, and homogenized (three presses) in 34% sucrose (density 1.128) containing 10 mM Tris-HCl (pH 7.4), 100 mM NaCl, and 1 mM EDTA (buffer A). The homogenate was centrifuged at 2000 rpm (500× *g*) for 5 min, and the supernatant was transferred into a fresh tube. We repeated the homogenization of the pellet once and repeated the centrifugation. The resultant supernatant was pooled with the first supernatant. The pooled supernatant, which represents the crude ROS, was centrifuged at 15,000 rpm (27,000× *g*) for 30 min to pellet the crude ROS. The pelleted ROS was resuspended in 48% sucrose (density 1.181) and poured into a clear Beckman centrifuge tube, followed by layering with 39% sucrose (density 1.146), 34% sucrose (density 1.128), and 28.7% sucrose (density 1.108). The gradient containing ROS was centrifuged at 25,000 rpm (113,000× *g*) for 90 min at 4 °C. We observed three distinct, well-separated bands on the gradient. Fraction 1 resolved between 28.7% and 34% sucrose, fraction 2 resolved between 34% and 39% sucrose, and fraction 3 resolved between 39% and 48%. The individual fractions were collected separately, diluted with buffer A, and centrifuged at 25,000 rpm for 30 min at 4 °C. The supernatant was decanted and the pellets were either resuspended in buffer A or directly used for the extraction of phosphoinositides. The fractions were also subjected to SDS-PAGE, followed by GelCode Blue staining to visualize proteins.

#### 2.2.2. Isolation of Bovine Retinal Pigment Epithelium

RPE was prepared from the bovine eyecup according to the method described [38,39] with modifications. Bovine eyes were dissected and the anterior segment and the neural retina were removed (used either for ROS preparation or frozen at −80 °C). Then, 1–2 mL of 0.32 M (10.9%) sucrose containing 10 mM Tris-HCl (pH 7.4) was added to each eyecup. The RPE in sucrose was gently brushed with a camel hair brush. The procedure was repeated once, pooling all of the brushed RPE in sucrose and centrifuging the RPE at 1100 rpm (150× *g*) for 10 min at 4 °C. The supernatant was discarded and the pellet was washed four times with 0.32 M sucrose, centrifuging each time as described above. These washing procedures were essential to remove adherent ROS and RBCs from the isolated RPE cells. The pellet was either directly lysed in PBS for the extraction of phosphoinositides or stored at −80 °C.

#### 2.2.3. Preparation of Bovine Cornea for the Isolation of Phosphoinositides

It is difficult to homogenize corneal tissue. We dissected the cornea, washed it in PBS, wrapped it in aluminum foil, and immediately dropped it into liquid nitrogen. The frozen cornea was pulverized in a mortar and pestle. The powder was dissolved in PBS and used to extract phosphoinositides.

#### 2.2.4. Protein-Lipid Overlay Assay

Membrane arrays (PIP-StripsTM) spotted with 100 pmol of 15 different biologically important lipids found in cell membranes were purchased from Echelon Research Laboratories (Salt Lake City, UT, USA) and used for protein-lipid overlay assay following the manufacturer’s instructions. The membranes were blocked with 3% (*w*/*v*) fatty acid-free bovine serum albumin (Sigma) in TBST (10 mM Tris [pH 8.0], 150 mM NaCl, and 0.1% [*w*/*v*] Tween 20) for 1 h at room temperature. Blocked membranes were incubated with 0.25 μg/mL GST or maltose-binding protein (MBP) fusion proteins containing 1D4 rhodopsin epitope overnight at 4 °C with gentle agitation. Then, the membrane was washed three times with TBST plus 3% fatty acid-free bovine serum albumin, after which the membrane was incubated with the anti-rhodopsin antibody (1:10,000) for 60 min at room temperature. The membrane was washed three times in TBST, followed by incubation with an HRPO-coupled anti-mouse secondary antibody (1:5000) for 60 min at room temperature. After washing, the membrane was developed with enhanced SuperSignal West Dura Extended Duration Substrate (Thermo Fisher Scientific, Waltham, MA, USA) and visualized using a Kodak Imager with chemiluminescence capability.

#### 2.2.5. Extraction of Phosphoinositides from Bovine Cornea, Retina, and RPE

Bovine cornea, RPE, and ROS were homogenized in PBS, and 2:1 chloroform/methanol was added to extract the PI. The extraction was repeated with chloroform to remove a majority of phospholipids. The chloroform layers were pooled into one tube, which represents the phospholipids (PLs). To the remaining solution, chloroform/methanol/HCl (2:4:0.1) was added to extract the phosphoinositides. The procedure was repeated with chloroform and HCl. Then, the chloroform layers were pooled into a clean tube, which represents the phosphoinositide (PI). The PL and PI pooled fractions were dried under nitrogen gas and the dried lipids were dissolved and sonicated in 150–200 µL of 1:9 chloroform/methanol. We measured lipid phosphorus from the PL and PI fractions according to the method described [40] and converted the measured lipid phosphorus concentration into phospholipid concentration [41]. Equal amounts of phospholipid (PL) from each sample were used for the measurement of phosphoinositide species.

#### 2.2.6. The Phosphoinositide ELISA Assay

The assays are based on the interaction of phosphoinositides with phospholipid-binding proteins as described [6] with some modifications. There are two types of domains that bind to PIs: FYVE and PH domains. These domains were cloned as either GST or MBP fusion proteins with a rhodopsin 1D4 epitope. The 2 × Hrs-FYVE domain was used to determine PI(3)P. Hrs is an early endosomal protein that is homologous to Vps27p and has an FYVE double zinc finger domain. The FAPP1 (the four-phosphate adapter protein-1 PH domain), TAPP1 (the tandem PH-domain-containing protein-1), PLCδ (the phospholipase C delta PH domain), and GRP1 (the PH domain of the general receptor for phosphoinositides isoform 1) PH domains were used to quantify levels of PI(4)P, PI(3,4)P2, PI(4,5)P2, and P1(3,4,5)P3, respectively [42].

The PI was measured using ELISA assay [6,43] by coating the isolated lipids on a 96-well Immulon 2HB plate in triplicate. The dried lipids were blocked with 3% BSA in PBS at room temperature. To detect PI(4)P, PI(3,4)P_2_, PI(4,5)P_2_, and PI(3,4,5)P_3_ PIs, the PI-binding proteins (FAPP1, TAPP1, PLCδ, and Grp1) were expressed as maltose-binding proteins (MBPs) with a rhodopsin 1D4 epitope. For PI(3)P detection, GST-2 × Hrs-ID4 fusion protein was used [6]. The MBP fusion proteins were purified on an amylose affinity chromatography column as described [44]. The purity of expressed fusion proteins was tested on SDS-PAGE, and the specificity of these PI-binding proteins towards specific PI was verified on lipid overlay assays [45]. These proteins were expressed in bacteria, and specific binding to the respective PIs was examined. The ELISA plate was incubated with the respective PI-binding protein (0.25–0.5 µg/mL) for 2 h at room temperature (RT), washed with PBS containing 0.05% Tween-20 at RT [43], and incubated with monoclonal rhodopsin 1D4 antibody at RT. After washing the plate, a mouse secondary antibody coupled to HRPO was added at RT. The plate was washed and the luminescence was detected using an ELISA plate reader. Relative luminescence units (RLUs) of each PI were normalized to phospholipid. For example, if we used 5 µL of sample for PI assay, the RLU would be divided by pmol of PL present in the 5 µL of the sample. The γ-axis shown in each figure is normalized RLU/pmol PL for each PI species.

#### 2.2.7. Recombinant Enzyme ELISA

Five pmol PI(4,5)P_2_ in phosphatidylcholine/phosphatidylethanolamine/phosphatidylserine (PC/PE/PS) solution was loaded into a 96-well ELISA plate in six triplicate wells. Control experiments were carried out with PC/PE/PS. The plate was allowed to air-dry for 2 h at RT. The six triplicate wells were divided into three groups, each group with two sets of triplicate wells. Group 1 was treated with only kinase buffer (1 mM EDTA, 50 mM Tris-HCl, 10 mM MgCl2); group 2 was treated with kinase buffer and PI3Kα (0.36 ng/µL); and group 3 was treated with kinase buffer, PI3Kα, and ATP (1 mM). All three treatment groups were incubated at RT for 30 min, and then washed 10× with PBST. The plate was then blocked with 3% BSA/PBS for 2 h at RT. After blocking, PI fusion protein PLCδ (1.0 µg/mL in 3% BSA/PBS) was added to one set of each group and the PI fusion protein Grp1 (1.0 µg/mL in 3% BSA/PBS) was added to one set of each group. The PI fusion proteins were incubated on the plate overnight at 4 °C. Following the overnight incubation, the plates were washed 10× with PBS-T and treated with rhodopsin 1D4 antibody (1.0 µg/mL in 3% BSA/PBS) for 2 h at RT. After incubation, the plate was washed again as before and treated with anti-mouse antibody (0.24 µg/mL) for 1 h at RT. After the 10× PBST wash step, the plate was treated with SuperSignal ELISA Femto Maximum Sensitivity Substrate, and the luminescence of the wells was measured using the BMG Labtech FLUOstar Omega microplate reader.

To evaluate the activity of PTEN phosphatase, 5 pmol PI(3,4,5)P3 was plated on a 96-well ELISA plate in six triplicate wells. Control experiments were carried out with PC/PE/PS. The plate was air-dried for two hours at RT. The six triplicate wells were divided into three groups, each with two sets of triplicate wells. Group 1 received no treatment, group 2 was treated with only reaction buffer (100 mM Tris-HCl, 10 mM DTT), and group 3 was treated with reaction buffer and PTEN (0.05 ng/µL). The plate was incubated with the treatment conditions for 1 h at RT and then washed 10× with PBS-T. After blocking the wells with 3% BSA/PBS for 2 h at RT, PI fusion protein Grp1 (1.0 µg/mL in 3% BSA/PBS) was added to one set of each group and the PI fusion protein PLCδ (1.0 µg/mL in 3% BSA/PBS) was added to one set of each group. The remainder of the experiment followed the antibody incubation, wash steps, and luminescence measurements described above.

### 2.3. Statistical Analysis

One-way analysis of variance (ANOVA) was used to determine statistical significance and a *p*-value < 0.05 is considered significant.

## 3. Results

### 3.1. Binding Specificity of PI-Binding Protein to Specific Phosphoinositide Species

**Figure 2:**
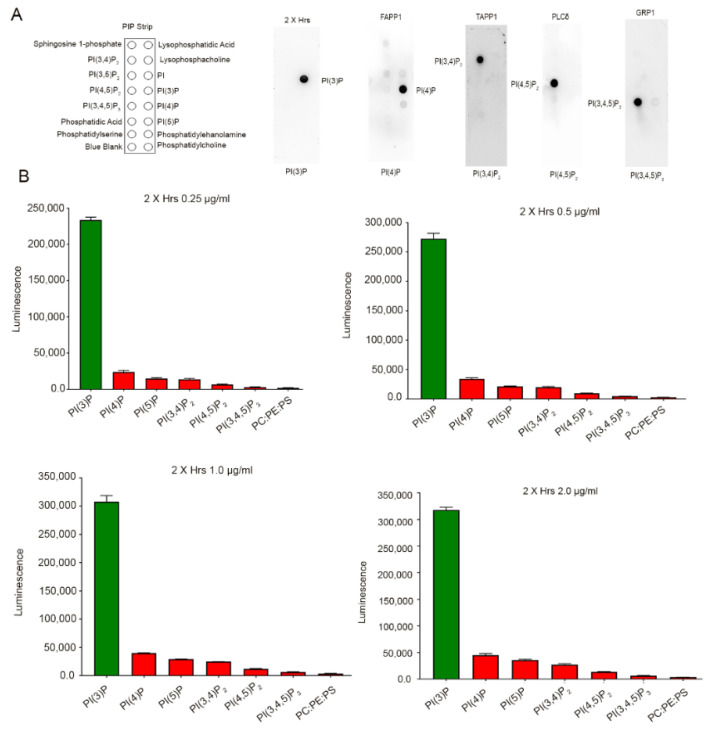
Protein-lipid overlay assay. In vitro protein-lipid overlay assay was carried out by incubating PIP-strips that were immobilized with 100 pmol of 15 lipids (**A**) with a purified PI-binding protein, 2× Hrs-1D4, FAPP1-1D4, TAPP1-1D4, PLCδ-1D4, and Grp1-1D4, followed by immunoblot analysis with the anti-rhodopsin antibody (**A**). The PI ELISA assay was carried out with different concentrations of 2× Hrs (0.25, 0.5, 1.0, and 2.0 µg/mL) using 2.5 pmols of PI(3)P, PI(4)P, PI(5)P, PI(3,4)P_2_, PI(4,5)P_2_, PI(3,4,5)P_3_, and PC/PE/PS (15:35:15) (**B**). The PI assay was carried as described in the methods. Data are mean ± SEM (*n* = 3).

To determine the binding specificity of the PI-binding proteins, an in vitro protein-lipid overlay assay was carried out by incubating PIP-strips that were immobilized with 100 pmol of 15 lipids (Figure 2A) with a purified PI-binding protein, 2× Hrs-1D4, FAPP1-1D4, TAPP1-1D4, PLCδ-1D4, and Grp1-1D4, followed by immunoblot analysis with the anti-rhodopsin antibody. The results indicated specific binding of 2× Hrs to PI(3)P, FAPP1 to PI(4)P, TAPP1 to PI(3,4)P_2_, PLCδ to PI(4,5)P_2_, and Grp1 to PI(3,4,5)P_3_ (Figure 2A). To determine the concentration of the fusion protein that gave maximum binding to the specific PI without any overlapping specificity with other PIs, we present the data for 2× Hrs (Figure 2B). The PI ELISA assay was carried out by coating 2.5 pmols of PI(3)P, PI(4)P, PI(5)P, PI(3,4)P_2_, PI(4,5)P_2_, PI(3,4,5)P_3_, and 100 pmol PC/PE/PS (50:35:15) carrier in which the PI lipids were prepared. The ELISA assay was carried out with four concentrations of 2× Hrs: 0.25 µg/mL, 0.5 µg/mL, 1.0 µg/mL, and 2.0 µg/mL. The results indicated a very specific binding of 2× Hrs to PI(3)P. In addition, increases in the 2× Hrs concentration resulted in increased binding (Figure 2B). The 2× Hrs fusion protein bound to other lipids at around <5%, further attesting to the specificity of 2× Hrs towards PI(3)P. Similarly, we carried out ELISA for other PI-binding proteins with maximum binding to specific lipids (data not shown).

### 3.2. Phosphoinositide Lipids in the Cornea

**Figure 3:**
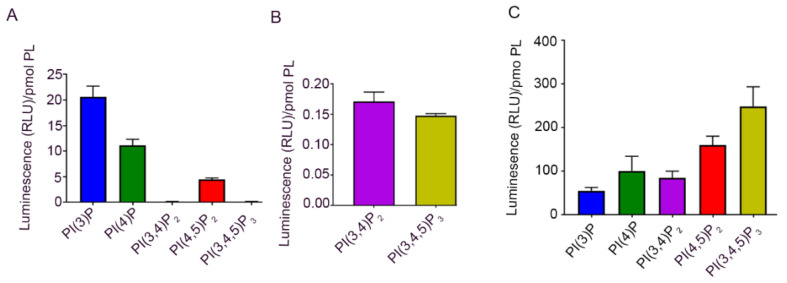
Identification and determination of the levels of PIs in the cornea and retinal pigment epithelium (RPE). The PI(3)P, PI(4)P, PI(3,4)P_2_, PI(4,5)P_2_, and PI(3,4,5)P_3_ levels were measured from the cornea (**A**). As the levels of PI(3,4)P_2_ and PI(3,4,5)P3 were low in the cornea, we plotted a graph on a different scale to show their presence in the cornea (**B**). The PI(3)P, PI(4)P, PI(3,4)P_2_, PI(4,5)P_2_, and PI(3,4,5)P_3_ levels were measured from the RPE (**C**). Data are mean ± SEM (*n* = 3).

To examine the PI species, we extracted phosphoinositides (PIs) from bovine cornea according to the methods described. We carried out PI ELISA and quantified PI(3)P, PI(4)P, PI-(3,4)P_2_, PI(4,5)P_2_, and PI(3,4,5)P_3_ using PI-binding proteins as described in the methods. The results indicated detectable levels of PI(3)P, PI(4)P, and PI(4,5)P_2_, and a very low level of PI(3,4)P_2_ and PI(3,4,5)P_3_ in the cornea (Figure 3A,B). Both PI(3,4)P_2_ and PI(3,4,5)P_3_ are formed by the action of class I PI 3-kinase (PI3K) [3]. There were no PIs in the bulk PL fraction extracted with chloroform/methanol (data not shown).

### 3.3. Phosphoinositide Lipids in the RPE

To examine the PI species, we extracted phosphoinositides (PIs) from bovine RPE according to the method described. We carried out PI ELISA and quantified PI(3)P, PI(4)P, PI(3,4)P_2_, PI-(4,5)P_2_, and PI(3,4,5)P_3_ using PI-binding proteins as described in the methods. The results indicated detectable levels of PI(3)P, PI(4)P, PI(3,4)P_2_, PI(4,5)P_2_, and PI(3,4,5)P_3_ in the RPE (Figure 3C). Among these PIs, the PI(3,4,5)P_3_ levels were highest in the bovine RPE [3].

### 3.4. Phosphoinositide Lipids in the Retina

**Figure 4:**
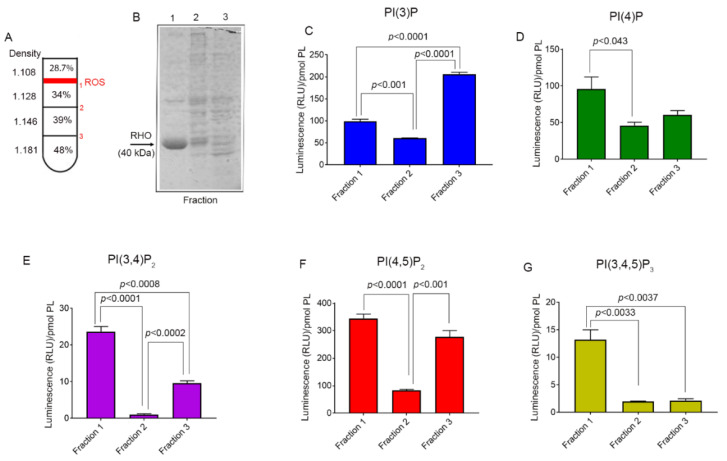
Identification and determination of the levels of PIs in the bovine retina. The bovine retina was subjected to sucrose density gradient centrifugation and three fractions were collected (**A**). Fractions collected from the sucrose gradients were subjected to SDS-PAGE (**B**). The arrow shows the presence of rhodopsin (40 kDa) in fraction 1. Each panel represents the levels of PI(3)P (**C**), PI(4)P (**D**), PI(3,4)P_2_ (**E**), PI(4,5)P_2_ (**F**), and PI(3,4,5)P_3_ (**G**). Data are mean ± SEM (*n* = 3). The significance is indicated on each panel.

The freshly obtained bovine retina was homogenized in 34% sucrose. The crude ROS was pelleted and resuspended in 48% sucrose. The crude ROS was then layered with 39%, 34%, and 28.7% sucrose (Figure 4A). Density gradient centrifugation resulted in three distinct layers, a layer between 28.7% and 34% (Fraction 1), a layer between 34% and 39% (Fraction 2), and a layer between 39% and 48% (Fraction 3). These three layers were individually collected, subjected to SDS-PAGE, and stained with GelCodeTM Blue stain (Figure 4B). Fraction 1 appears to be rod outer segments with the presence of rhodopsin signature, which is absent from fractions 2 and 3. Fractions 2 and 3 may represent the broken inner segments and other retinal cell types.

To examine the PI species, we extracted phosphoinositides (PIs) from the three fractions according to the method described in the methods. We carried out PI ELISA and quantified PI(3)P, PI(4)P, PI(3,4)P_2_, PI(4,5)P_2_, and PI(3,4,5)P_3_ using PI-binding proteins as described in the methods. The results indicated detectable levels of PI(3)P, PI(4)P, PI(3,4)P_2_, PI(4,5)P_2_, and PI(3,4,5)P_3_ in ROS fraction 1 (Figure 4C–G). The other fractions presumably representing other cell types of the retina also contain variable amounts of these PI species (Figure 4C–G).

**Figure 5:**
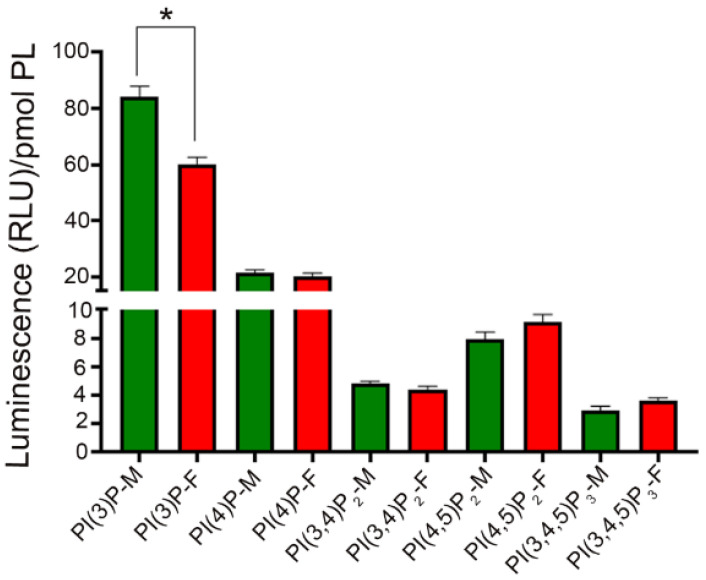
Determination of the levels of PIs in the mouse retina. Phosphoinositides were extracted from male (M) and female (F) Balb/c mouse retinas. The PI lipids were coated on the ELISA plate and we measured PI(3)P, PI(4)P, PI(3,4)P_2_, PI(4,5)P_2_, and PI(3,4,5)P_3_ levels using PI-binding proteins as probes. Data are mean ± SEM (*n* = 3). * *p* < 0.01, the significance of PI(3)P levels between male and female mouse retina. RLU, relative luminescence units.

To relate the measurements taken in the bovine eye with a much smaller mouse eye, phosphoinositides were extracted from male and female Balb/c mouse retinas and subjected to PI assays, as described in the methods section. The results indicated that all five tested PIs were present in the mouse retina (Figure 5). We found a significant difference in the level of PI(3)P in female mouse retina compared with male mouse retina (Figure 5). There was no sex difference in the levels of PI(4)P, PI(3,4)P_2_, PI-4,5-P_2_, and PI(3,4,5)P_3_ (Figure 5). The level of PI(3)P in bovine tissue was comparable to that in mouse tissue. The levels of PI(4)P, PI(3,4)P_2_, and PI(4,5)P_2_ were lower in mouse tissue than in bovine tissue. The level of PI(3,4,5)P_3_ in mouse tissue was slightly lower than bovine tissue. The mouse data suggest that the PIs can be easily measured with ELISA using PI-binding proteins as probes.

### 3.5. Recombinant PI3K and PTEN Enzyme ELISA

**Figure 6:**
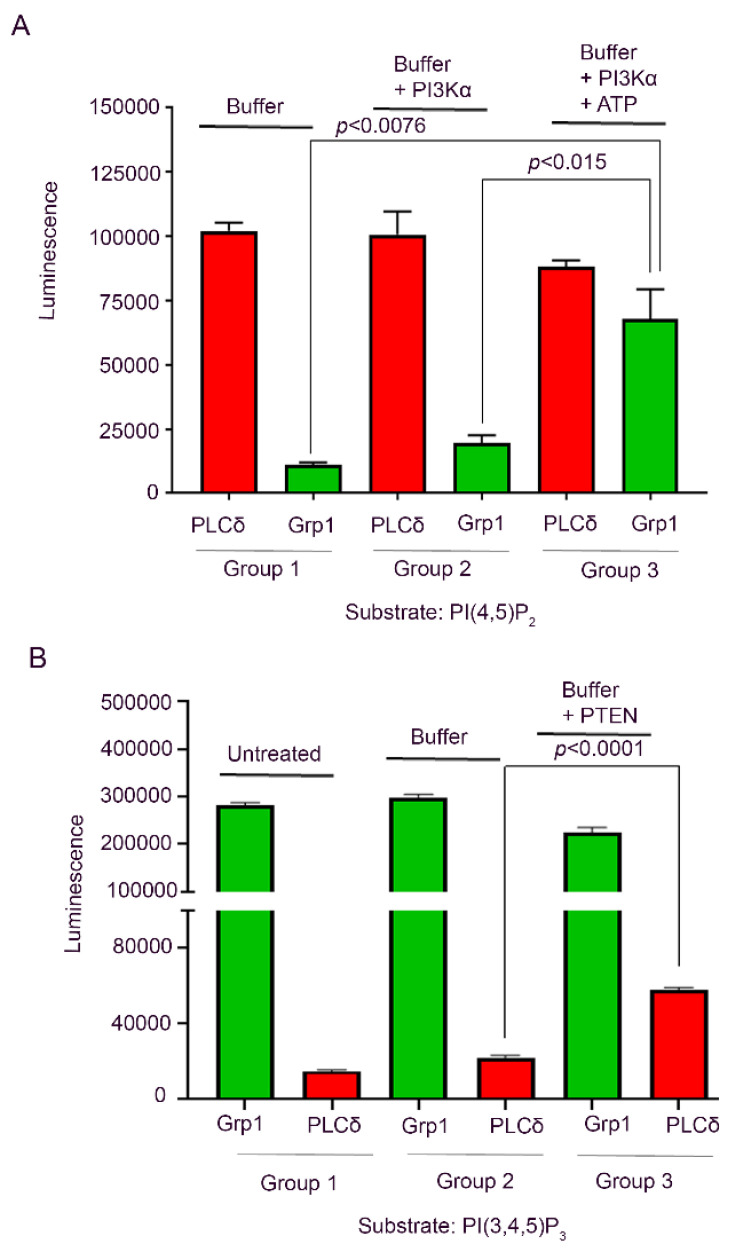
Recombinant PI kinase and PI phosphatase ELISA. For PI kinase assay, 5 pmol PI(4,5)P_2_ in PC/PE/PS solution was loaded into a 96-well ELISA plate and divided into three groups. Group 1 is treated with only kinase buffer; group 2 with kinase buffer and PI3Kα; and group 3 with kinase buffer, PI3Kα, and ATP (**A**), and carried out phosphorylation and PI assay as described in the methods. In each group, PI(4,5)P_2_ and PI(3,4,5)P_3_ were detected using the PH domains of PLCδ and Grp1, respectively. For PI phosphatase, PTEN 5 pmol PI(3,4,5)P_3_ was plated on a 96-well ELISA plate and divided into three groups. Group 1 is untreated, group 2 with only reaction buffer, and group 3 with reaction buffer and PTEN (**B**). The phosphatase treatment and the PI assay was carried out as described in the methods. In each group, PI(3,4,5)P_3_ and PI(4,5)P_2_ and were detected using the PH domains of Grp1 and PLCδ, respectively. Data are mean ± SEM, (*n* = 3). Significance is indicated in the figure.

In this study, we examined the phosphorylation of PI(4,5)P_2_ by PI3K and dephosphorylation of PI(3,4,5)P_3_ by 5′phosphatase, PTEN on the ELISA plate. The PI(4,5)P_2_ and PI(3,4,5)P_3_ were detected using PH domains of PLCδ and Grp1, respectively. Our data indicate that incubation of PI(4,5)P_2_ with PI3K and ATP resulted in the generation of PI(3,4,5)P_3_ (Figure 6A). The PTEN phosphatase experiment suggests that the incubation of PI(3,4,5)P_3_ with PTEN resulted in the generation of PI(4,5)P_2_ (Figure 6B).

## 4. Discussion and Conclusions

In all mammalian cells, all seven phosphoinositides are formed [46]. Their formation is also present in the retina/photoreceptor cells based on the PI enzymatic assays using PIs as substrates [47,48,49,50]. In rod photoreceptor cells, the class III PI3K product PI(3)P formation is light-dependent; however, the other class I PI3K-generated products were not detectable [6]. The PI signals play an important role in various cellular processes [1], including host–pathogen interactions [20,21,22]. In the present study, we examined five distinct phosphoinositides using PI-binding proteins that detect PI(3)P, PI(4)P, PI(3,4)P_2_, PI(4,5)P_2_, and PI(3,4,5)P_3_ as probes in ocular tissues/cells. We were not able to examine PI(5)P and PI(3,5)P_2_. The PI-binding protein probes for these two PIs are difficult to express as soluble proteins in bacteria, and they have some overlapping binding with other PIs (data not shown).

The cornea is a sensitive tissue that is susceptible to fungal, bacterial, and viral infections [51]. These infections cause a series of inflammatory reactions, which can ultimately lead to blindness. Phosphoinositides regulate several key cellular processes, which include actin polymerization, vesicular trafficking, and cell survival [1]. Further, phosphoinositides play an important role in host–pathogen interactions, and these pathogens hijack PI signaling during infection [20,21,22]. The intracellular microbial pathogens deploy PI metabolism to facilitate their uptake by host cells for their survival and replication [20]. In rabbit corneal epithelium, an active inositol polyphosphate and phosphoinositide metabolism has been reported [5]. This study for the first time showed the activities of diacylglycerol kinase, PI kinase, PIP kinase, and inositol phosphatase [5], suggesting that PI metabolism may play a role in corneal biology. Further studies are needed to examine whether PIs have any role in the proliferation of corneal epithelial cells and their differentiation.

With the advent of PI-binding fusion proteins, we were able to detect the PIs in the cornea, RPE, and retina. The pattern of these PIs was observed to be different in the different tissues. For example, PI(3)P, PI(4)P, and PI(4,5)P_2_ levels were higher than levels of PI(3,4)P_2_ and PI(3,4,5)P_3_ in the cornea. Both PI(3,4)P_2_ and PI(3,4,5)P_2_ were generated from the action of the class I PI3K [3]. We previously reported that corneal cells infected with Adenovirus 19 utilized class I PI3K/Akt activation for viral pathogenesis [23]. Both PI(3,4)P_2_ and PI(3,4,5)P_3_ lipids are known to activate Akt [3,52]. These observations suggest that, under physiologic conditions, the cornea has very low levels of class I PI3K, which might be upregulated in infection states for host–pathogen interaction. The low levels of these two PIs could be substrates of 3′PI phosphatase, PTEN, which dephosphorylates phosphate from the third position to generate PI(4)P from PI(3,4)P_2_ and PI(4,5)P_2_ from PI(3,4,5)P_3_.

In this study, we were able to measure the relative luminescence and normalized to phospholipid content in the extracted phosphoinositide fraction. Recently, Wensel’s Laboratory has reported that, in rod, outer segments and fragments of inner segments contain PI(3)P at 0.0035 mol% and PI(4)P as well as PI(4,5)P_2_ in the order of 0.04 mol% (10-fold higher than the levels of PI(3)P) of total phospholipid [13]. The PI measurements reported in this manuscript will help to monitor changes in the levels of PI species relative to control. This will enable comparisons of relative levels between wild type mice and knockout mice or various disease models, for example. This may allow for investigations into the role of various PIs in different disease conditions.

A recent study showed that PI(3)P lipid is essential for RPE functions [11]. Our PI data showed that bovine RPE cells have all five PIs tested here, and PI(3,4,5)P_3_ levels were higher than levels of the other PIs. Interestingly, retinal degeneration has been observed in mice lacking PTEN in the RPE [53]. These observations suggest that PI(3,4,5)P_3_ levels must be regulated in the RPE for the health and maintenance of RPE cells.

Our PI data on the retina show differential levels of PIs in three fractions isolated from the sucrose density gradient centrifugation. Interestingly, the ROS fraction contains all of the PIs tested in this study. However, the levels of the PI3K-generated PIs, PI(3,4)P_2_ and PI(3,4,5)P_3_, were the lowest in this fraction. An earlier study conducted on mouse ROS failed to detect these two PIs [6]. This failure could be because of lower levels of these PIs in mouse ROS than in bovine ROS. The decreased levels of PI(3,4)P_2_ and PI(3,4,5)P_3_ could also be the result of increased PTEN activity. Consistent with this notion, deletion of PTEN in a retinitis pigmentosa mouse model halted the death of cone photoreceptor cells [54]. Our lab previously reported the localization of class I PI3K subunits in isolated rod outer segment membranes, and PI3K activity was associated with phosphotyrosine and insulin receptor immunoprecipitates [55]. The current study on bovine ROS showed the presence of class I PI3K-generated products PI(3,4)P_2_ and PI(3,4,5)P_3_ to further confirm our earlier studies [55]. We also found increased levels of PI(4)P and PI(4,5)P_2_ in the ROS compared with the other fractions, and we reported the PI enzymes that make PI(4)P and PI(4,5)P_2_ in bovine ROS [3]. Consistent with our previous observations, these findings suggest that an active PI cycle is present in the rod outer segments.

Changes in the expression or activity of phosphoinositide kinases and phosphatases have been implicated in various diseases, including retinal degeneration, myopathy, diabetes, and cancer. The recombinant PI kinase and PI phosphatase ELISA assays employed in this study would help to screen PI kinases and PI phosphatases that either activated or downregulated in disease conditions, by incubating protein lysates from the healthy and degenerated retina with various PIs. The PI-binding proteins will be applicable to screen novel therapeutic agents for the management of anomalous phosphoinositide metabolism.
